# Reliability and validity test of a novel three-dimensional acetabular bone defect classification system aided with additive manufacturing

**DOI:** 10.1186/s12891-022-05365-y

**Published:** 2022-05-09

**Authors:** Jingwei Zhang, Yi Hu, Hua Ying, Yuanqing Mao, Zhenan Zhu, Huiwu Li

**Affiliations:** Shanghai Key Laboratory of Orthopaedic Implants, Department of Orthopaedic Surgery, Shanghai Ninth People’s Hospital, Shanghai Jiaotong University School of Medicine, 639# Zhizaoju Road, Shanghai, 200011 People’s Republic of China

**Keywords:** Three-dimensional, Classification, Hip revision arthroplasty, Acetabular bone defect, Additive manufacturing

## Abstract

**Background:**

Accurate assessment of acetabular defects and designing precise and feasible surgical plans are essential for positive outcomes of hip revision arthroplasty. Additive manufacturing (AM) is a novel technique to print physical object models. We propose a three-dimensional acetabular bone defect classification system aided with AM model, and further assess its reliability and validity under blinded conditions.

**Methods:**

We reviewed 104 consecutive patients who underwent hip revision arthroplasty at our department between January 2014 and December 2019, of whom 45 had AM models and were included in the reliability and validity tests. Three orthopedic surgeons retrospectively evaluated the bone defects of these 45 patients with our proposed classification, made surgical plans, and repeated the process after 2 weeks. The reliability and validity of the classification results and corresponding surgical plans were assessed using the intra-class correlation coefficient or kappa correlation coefficient.

**Results:**

The reliability and validity of the classification results were excellent. The mean initial intra-class correlation coefficient for inter-observer reliability was 0.947, which increased to 0.972 when tested a second time. The intra-observer reliability ranged from 0.958 to 0.980. Validity of the classification results also showed a high kappa correlation coefficient of 0.951–0.967. When considering corresponding surgical plans, the reliability and validity were also excellent, with intra-class correlation coefficients and kappa correlation coefficients measuring all over 0.9.

**Conclusions:**

This three-dimensional acetabular defect classification has excellent reliability and validity. Using this classification system and AM models, accurate assessment of bone defect and reliable surgical plans could be achieved. This classification aided with AM is a promising tool for surgeons for preoperative evaluation.

## Introduction

The number of hip revision arthroplasty is predicted to increase by 70% from 2014 to over 85,000 by 2030 [1]. In these cases, the acetabulum is always defective due to aseptic loosening, periprosthetic osteolysis and infection, and the remaining bone quality and stock vary. Managing acetabular bone loss, especially accurately assessing defects and designing precise and feasible surgical plans, has increasingly become a challenge for orthopedic surgeons [2].

Acetabular defect classifications are introduced to guide preoperative evaluation, including those by Paprosky et al. [3], the American Academy of Orthopaedic Surgeons(AAOS) [4], Engh et al. [5] and Gross et al. [6]. However, there are limitations to these classifications. The Paprosky, AAOS, and Gross classifications were reported to have poor reliability when used by surgeons [7]. The Engh and Gross classifications are intended to simplify the AAOS classification to improve reproducibility and communication. However, this is done at the expense of accurate preoperative determination of prosthesis and bone graft material to be used, and the reliability and validity of these two systems was still poor [8]. The Paprosky classification is based on available reconstructive strategies and is widely used in clinical practice, but there remains dispute over its reliability and validity [9]. Campbell et al. tested the intra- and inter-observer reliability of the Paprosky classification. They found that only the originators of the classification had moderate intraobserver reliability, with kappa values over 0.75. For experts and residents, the intraobserver agreement was poor, with kappa values less than 0.5. It became worse when considering interobserver reliability with kappa values less than 0.4 [7]. Yu et al. also found moderate interobserver agreement with kappa values of approximately 0.56, if there were no teaching sessions [10]. In addition, Yu et al. reported that the Paprosky classification was less valid in evaluating defects in the posterior acetabular wall or ischium, as a result of the radiopaque acetabular cup obscuring these features on standard anteriorposterior radiographs of the pelvis [10]. Some researches have concluded that the Paprosky classification can be subjective and should be considered only as a general guide [7, 11]. Generally, current classification systems are more useful in the evaluation of simple acetabular defects than of complex defects, with few accurately guiding surgical plans [8, 12].

One possible reason for this is that the currently used classifications, proposed in the 1990s, are limited by outdated radiological techniques. They are mainly dependent on two-dimensional (2D) X-rays which only provide general anatomical clues [12–15] and do not accurately represent three-dimensional (3D) structures. With the development of additive manufacturing (AM) technology, standard 3D-computed tomography (CT) images are now used to produce physical models of the patient’s anatomy. Physicians can use these models to accurately obtain key information, which might be difficult to obtain through traditional images alone and, therefore, can simulate various surgical plans and design implants [16]. AM models have proven to be more advantageous in assessing bone defects and designing surgical plans in complex anatomical areas and revision cases than X-ray or CT scans [16–20].

Our joint reconstruction department has used AM models for preoperative evaluation and surgical planning for patients with acetabular defects requiring revisions since 2007. In this study, we propose a 3D acetabular defect classification system aided with AM models and its corresponding surgical approaches, and test its reliability and validity.

## Methods

### Patients enrolment and AM model creation

This study was approved by the Ethics Committee of our hospital. We reviewed 104 patients who underwent hip revision arthroplasty at our department between January 2014 and December 2019 for aseptic acetabular prosthesis loosening or osteolysis. Anteroposterior and lateral radiographs (RAX, Siemens, Erlangen, Germany) of the affected hip were obtained. The acetabular defects detected by X-rays required additional CT scans (SOMATOM Definition Flash, Siemens, Erlangen, Germany) covering the bilateral anterior superior iliac spine and the posterior borders of the medial and lateral condyles with 0.5-mm interspacing thickness for more specific examination. If severe defects were detected on CT, AM model was employed. Severe defects were defined as those which might hamper the commercial prosthesis placement or affect initial stability and require augments during the operation [20, 21]. AM models were created for the 45 patients diagnosed with severe defects for preoperative planning. Surgeries was performed by the same group of experienced qualified surgeons. The intraoperative findings and surgical treatments used were considered the gold standard for comparison with the subsequent assessment by surgeons. Surgeries were successful, and no re-revisions have been reported at the time of this writing. These 45 patients were included in this study for reliability and validity testing.

AM is used to convert CT scans into isometric physical object models [16]. The CT results were imported into Mimics (version 19.0, Materialise, Belgium) to rebuild the CT model. AM models were prepared using photosensitive resin by stereolithography technology (Lite450, UnionTech, China). The AM model had a resolution of 0.1 mm and required 24 h for painting and cost approximately $470 or $780 for the hemi- or whole pelvis, respectively.

### Classification system

Based on our experience, we propose a 3D acetabulum defect classification system as follows:

- Type I: There are no obvious or minor acetabular defects. The initial rotational and vertical stability of the cup can be provided by the host bone.
- Type II: There is sufficient bone mass in the anterosuperior acetabulum, ischial ramus, and pubic ramus, while defects exist in the stress-bearing posterosuperior acetabulum. The host bone provides only rotational stability to the cup.
- Type III: There are defects in the anterosuperior acetabulum, ischial ramus, or pubic ramus. Both initial rotational and vertical stabilities are lost.
- Type IV: Severe destruction of acetabular structures with high risk of pelvic discontinuity is observed.

### Corresponding surgical plans

**Fig. 1:**
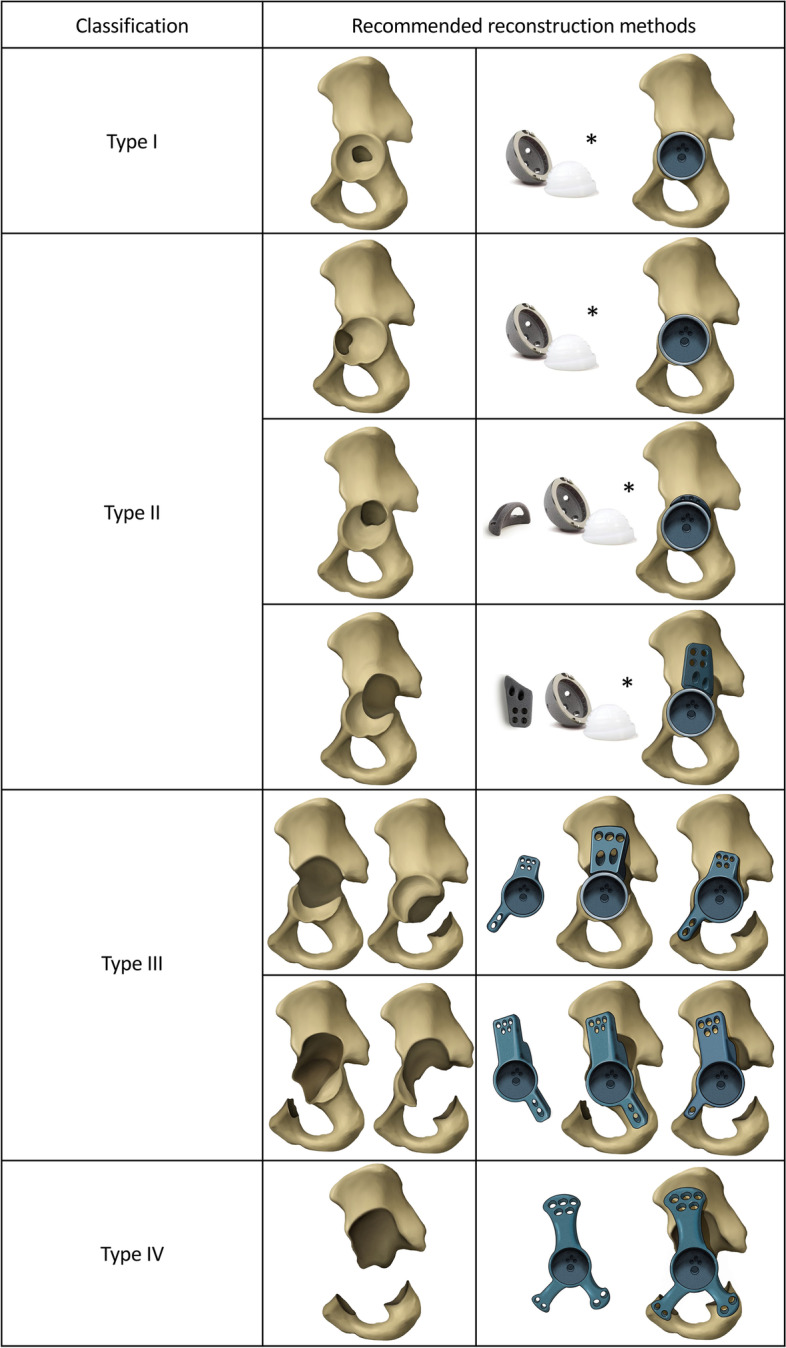
Illustration of the classification system and possible reconstruction methods. *, the prosthesis from Zimmer (Zimmer Biomet, Warsaw, Indiana, USA) was used as example, and permission was obtained

We suggest the surgical plans outlined below as solutions based on our experience. However, it must be noted that these are not the only viable solutions (Fig. 1).

**Fig. 2:**
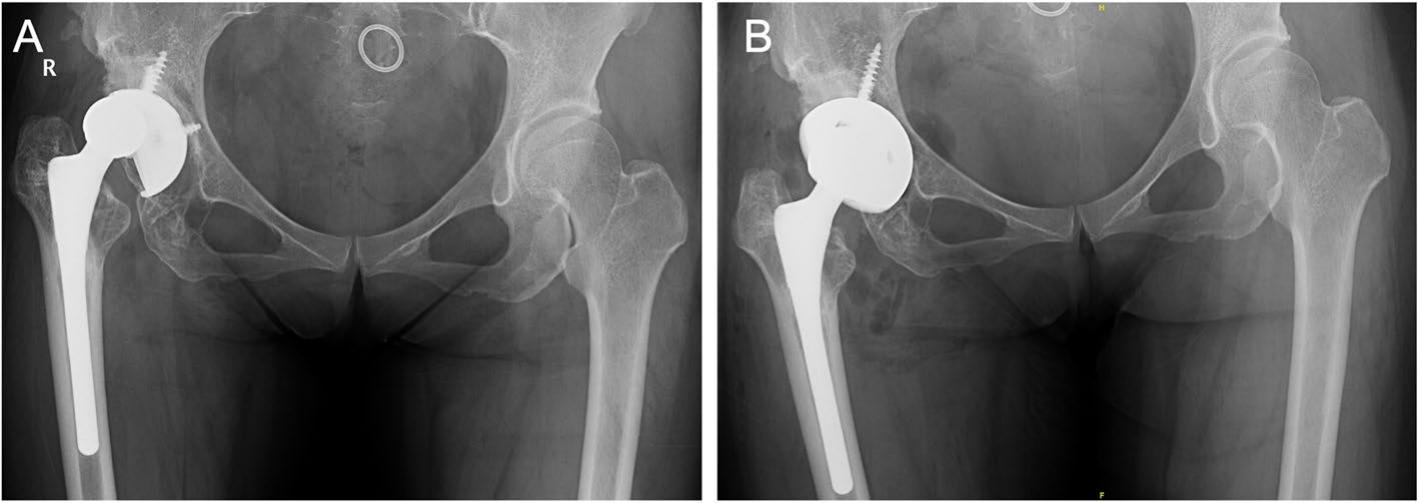
**A** A 47-year-old female was diagnosed with right hip prosthetic aseptic loosening with small defects in acetabulum but an intact acetabular ring 13 years after THA. **B** A commercial cup was used

For type I, an intact acetabular ring was obtained by slight drilling. A commercial cup was used (Fig. 2).

**Fig. 3:**
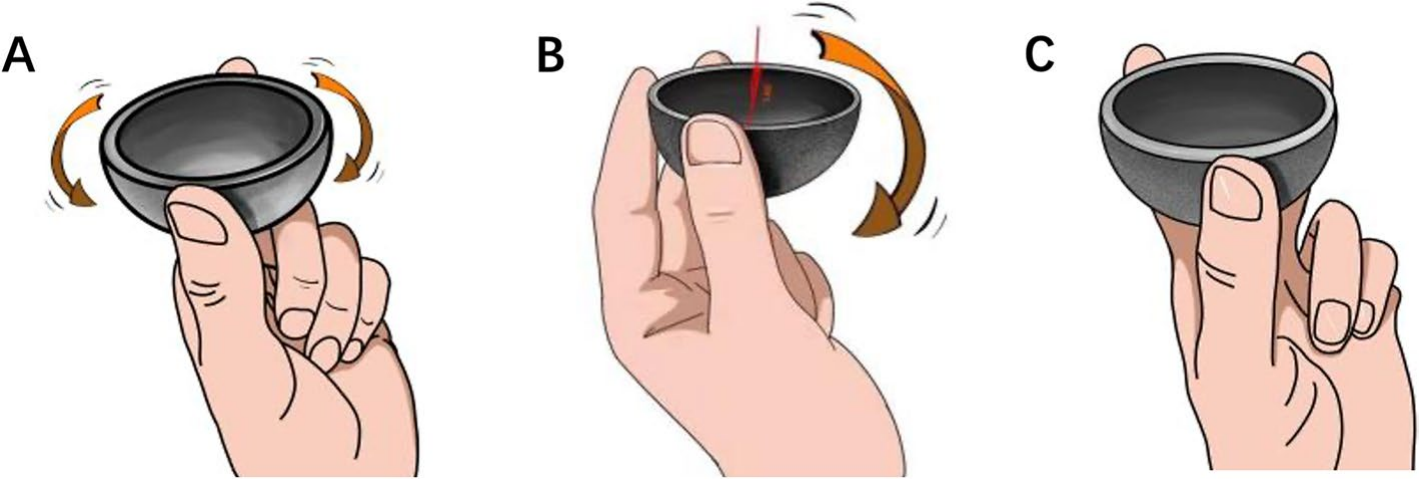
Illustration of the three-point fixation spanning over 180 degrees. **A** the cup rotates when only fixed by two points; **B** the cup inclines to the unsupported side when fixed by three points spanning within 180 degrees; **C** three points spanning over 180 degrees hold the hemisphere cup firmly

For type II, the rotational stability of the cup was provided by a three-point fixation spanning over 180° (Fig. 3). Depending on bone defects and vertical stability, surgical plans were divided into three detailed situations:

1. With no obvious defects in the stress-bearing posterosuperior acetabulum, initial rotational and vertical stabilities were obtained. A commercial cup was used (Fig. 4).

**Fig. 4:**
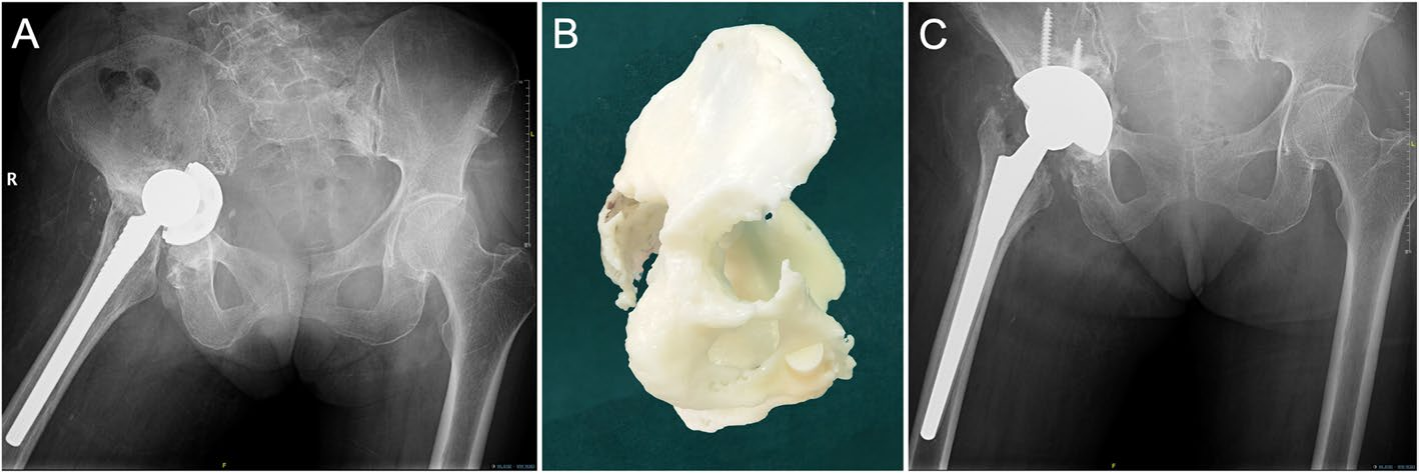
**A** A 77-year-old female was diagnosed with right hip prosthetic aseptic loosening. **B** Am model showed defects in the anteroinferior acetabulum. The anterosuperior acetabulum, the ischial ramus, the pubic ramus and stress-bearing posterosuperior acetabulum were intact. **C** Initial vertical and rotational stability was achieved with a commercial cup
2. With cavity defects in the stress-bearing acetabulum, vertical stability was achieved by posterosuperior augments. Because commercial augments were sufficient to achieve effective fixation in cavity defects, they were treated using a commercial cup with commercial augments (Fig. 5).

**Fig. 5:**
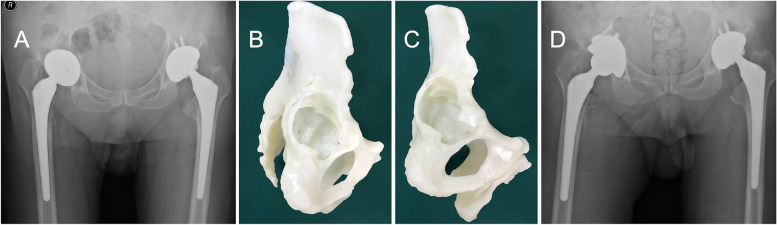
**A** A 74-year-old male was diagnosed with right hip prosthetic aseptic loosening 4 years after THA. **B**-**C** Am model showed defects in the anteroinferior acetabulum and cavity defects in the posterosuperior. The vertical stability was relied on the reconstruction of the stress-bearing acetabular region by commercial augments. **D** Commercial augments and a cup were used to reconstruct the right acetabulum
3. With uncontained defects in the stress-bearing region, the posterosuperior acetabulum was damaged in a plain wall. The shear force would be quite large when only commercial augments were used. Therefore, customized augments with fixed hooks or buttress plates were necessary to avoid a large shear force (Fig. 6).

**Fig. 6:**
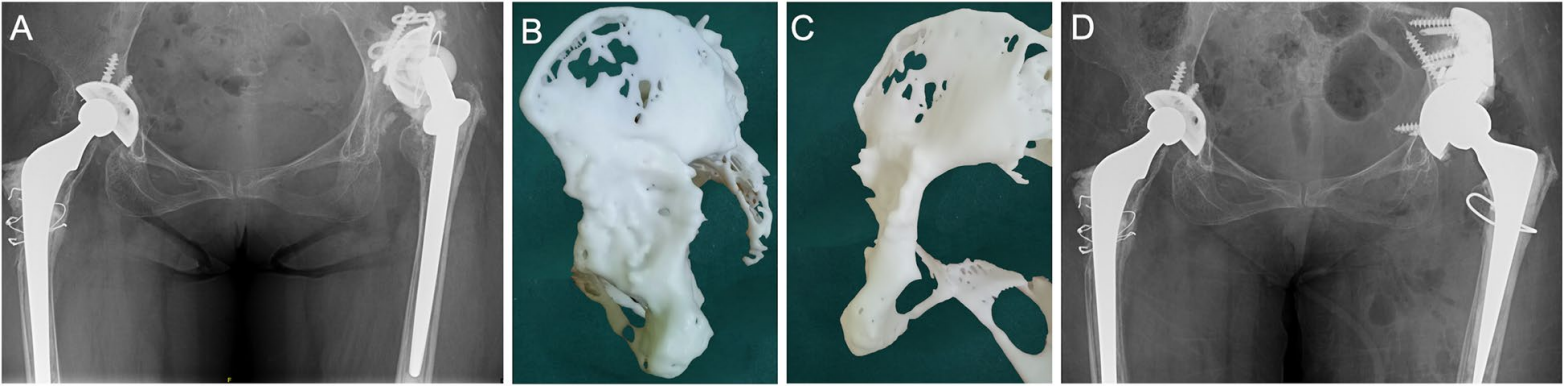
**A** A 62-year-old female was diagnosed with left hip prosthetic aseptic loosening 20 years after THA. **B**-**C** Am model showed uncontained defects in the posterosuperior acetabulum. The vertical stability could be achieved by reconstructing the posterosuperior stress-bearing acetabulum with augments with wing. **D** A customed augment with a wing was used to reconstruct the left acetabulum with a commercial cup

**Fig. 7:**
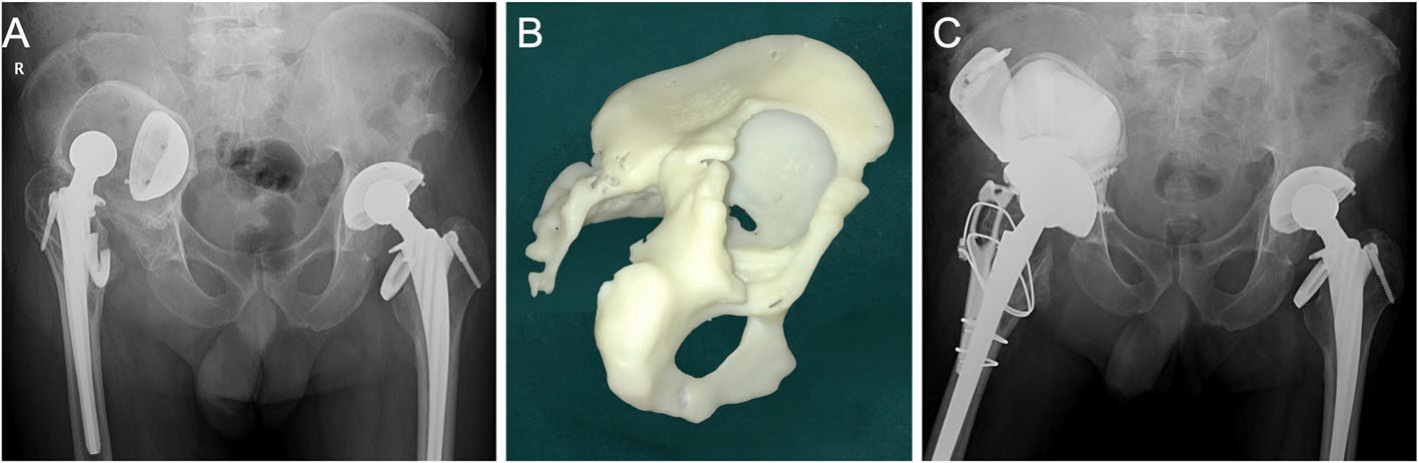
**A** A 68-year-old male was diagnosed with right hip prosthetic aseptic loosening 20 years after THA. **B** AM model showed that the entire superior acetabulum was defective while the ischial ramus and the pubic ramus was intact. The remaining bone was difficult to support the rotational and vertical stability of the cup. An augment was designed to rebuild the superior acetabulum. The vertical stability could be achieved and the rotational stability could be ensured by clamping the cup with the superior augment, the ischial ramus and the pubic ramus. **C** Acetabulum reconstruction was made by three customized augments reinforcing the right acetabulum and a commercial cup

**Fig. 8:**
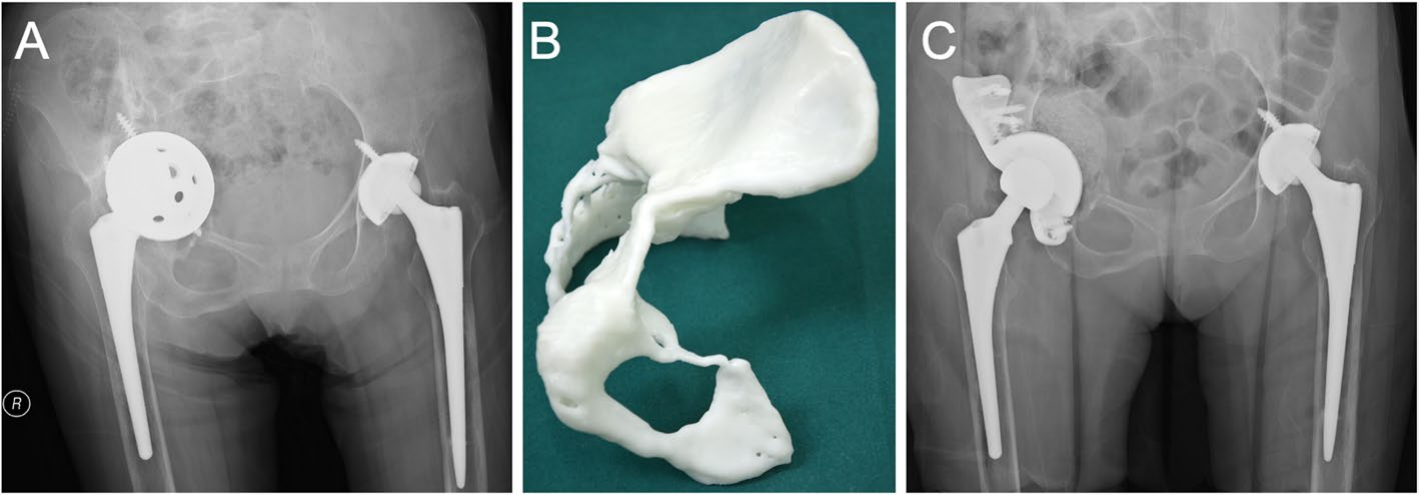
**A** A 60-year-old female was diagnosed with right hip prosthetic aseptic loosening 1 year after right hip revision. **B** AM model showed the inferior, superior and anterior acetabulum and the ischial ramus was defected. The vertical and rotational stability of the cup cannot be obtained and it was difficult to reconstruct one place by augments to obtain a firmly clamped cup. **C** A customized cage was used. Rotational stability was obtained with cage wing fixed into the ala of ilium and the ischial ramus by screws. Vertical stability was ensured by the acetabular cup directly contacting the superior host bone

For type III, when the anterosuperior acetabulum or ischial ramus was defective, a buttress plate or customized augmentation is used to repair it. After that, the cup was secured by a three-point fixation over 180°, together with the pubic ramus. A cup-cage or customized cage can also be used as an alternative (Fig. 7). When more than two of these three structures were defective, three-point fixation was not possible, further necessitating the need for a cup-cage or customized cage (Fig. 8).

**Fig. 9:**
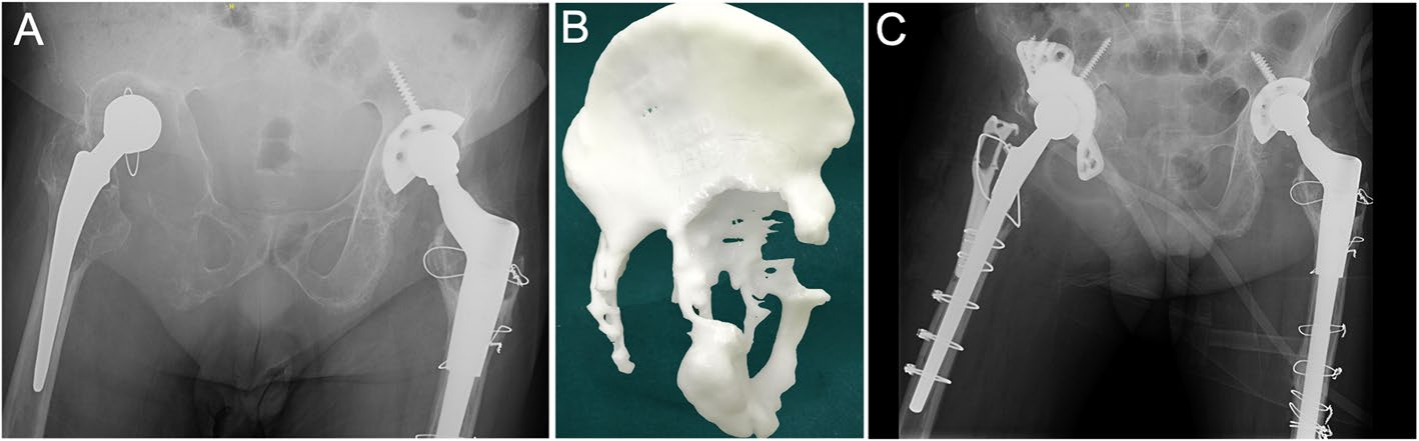
**A** A 54-year-old male was diagnosed with right hip prosthesis loosening 24 years after THA [20]. **B** AM model showed severe osteolysis around the acetabulum in the superior and inferior acetabulum, ischial ramus and pubic ramus. Some cortical bone remained in the ischial ramus on AM model, but it was proved to be radiopaque bone cement in operation and the ischial ramus was completely destroyed (We had reported this specific intraoperative finding and other misleading cases under AM models in another study in detail [20]. The vertical and rotational stability could not be obtained. **C** Although the operation was managed with a customized cage, we believed that the initial stability was insufficient because the cage was fixed to the ala of ilium only by a screw in the superior. Such patients might benefit more from customized hemipelvic prosthesis [20]

For type IV, a customized hemipelvic prosthesis was required (Fig. 9).

### Reliability and validity test and data analysis

We calculated the sample size required before the reliability test and set the intra-class correlation coefficient (ICC) at 0.95 with a confidence interval length ranging from 0.07 to 0.10, using PASS 15.0 (NCSS, Kaysville, Utah, USA). It was shown that a sample size of at least 20 to 36 reached confidence level of 95%. For validity tests using kappa (κ) correlation confidence, it is suggested that sample size assume no fewer than 20 and preferably at least 25–50 rated cases [22]. Above 45 patients were evaluated for reliability and validity test. Three experienced surgeons qualified to perform joint revisions were asked to use the classification proposed above to evaluate the bone defects and construct surgical plans independently and retrospectively for all enrolled 45 patients. The surgeons had access to X-ray, CT and AM models. The evaluations were performed blind, with surgeons possessing knowledge of only the patient identification number. Classification results and corresponding surgical plans were recorded. This process was repeated after two weeks. And the sequence of the patients was disrupted. The classification results and corresponding surgical plans for all the 45 patients were assessed for reliability and validity. Intra-observer reliability is the agreement between the same observer on separate occasions. Agreement between all observers is referred as inter-observer reliability. Additionally, the validity was assessed by comparing the proposed classification results and surgical plans to surgical records. Statistical analyses were performed using MedCalc (version 18.0, MedCalc Software Ltd, Ostend, Belgium). The ICC was used to reflect inter- and intra-observer reliability, and the κ correlation coefficient was used to reflect validity. The mean value and 0.95 confidence interval were used. ICC values are interpreted as follows: values ≤ 0.50 as poor, 0.50–0.75 as moderate, 0.75–0.90 as good, and above 0.90 as excellent [23]. And kappa values are interpreted as follows: values ≤ 0.40 as poor, 0.40–0.59 as fair, 0.60–0.74 as good, and 0.75–1.00 as excellent indicating almost perfect agreement [24].

## Results

According to the proposed classification system, 30 patients met the requirements for Type II, 9 for type III, and 6 for type IV classification, respectively.

The ICC and κ values for the reliability and validity tests of the classification results were all excellent (Tables 1 and 2). The mean initial inter-observer ICC was 0.947, which increased to 0.972 in the second test (Table 1). The intra-observer ICC values for three individual surgeons ranged from 0.958 to 0.980. The mean κ value for the validity of classification results was 0.951–0.967 (Table 2).

**Table 1:** The ICC values of inter-observer reliability

	Classification results	Surgical plans
1^st^ Test	0.947 (0.915–0.969)	0.960 (0.936–0.976)
2^nd^ Test	0.972 (0.955–0.984)	0.968 (0.948–0.981)

**Table 2:** The ICC values of intra-observer reliability for classification results and mean κ values for validity

	The ICC values	The κ values
Surgeon 1	0.980 (0.964–0.989)	0.952
Surgeon 2	0.958 (0.925–0.977)	0.967
Surgeon 3	0.980 (0.964–0.989)	0.951

When considering detailed surgical plans, excellent ICC and κ values were also observed (Tables 1 and 3). The mean initial inter-observer ICC was 0.960, which increased to 0.968 when tested again (Table 1). The intra-observer ICC values for the three surgeons were 0.988, 0.964, and 0.987, respectively. The κ values for validity of detailed surgical plans were also high, all exceeding 0.920 (Table 3).

**Table 3:** The ICC values of intra-observer reliability for surgical plans and mean κ values for validity

	The ICC values	The κ values
Surgeon 1	0.988 (0.978–0.993)	0.943
Surgeon 2	0.964 (0.935–0.980)	0.922
Surgeon 3	0.987 (0.976–0.993)	0.929

The ICC values were over 0.9, indicating that the classification results and surgical plans had excellent intra- and inter-observer reliability. The κ values were all larger than 0.75, showing that the classification results and surgical plans had excellent validity compared with surgical records.

## Discussion

Accurate assessment of acetabular defects and designing precise and feasible surgical plans are critical for ensuring successful surgeries [25]. Acetabular defect classifications are introduced to guide preoperative evaluation. However, current classification systems have several limitations [5, 7, 8, 26–30]. Campbell et al. [7] assessed the reliability of three major acetabular classifications described by Paprosky et al. [3], AAOS [4] and Gross et al. [6]. They reported a moderate range of intra-observer agreement in the innovator group, and a comparatively lower agreements in the non-innovator group. Gozzard et al. [27] reported that the Paprosky classification system achieved moderate to good levels of agreement, but the bone stock loss classification systems were inconsistent and unreliable, which prevented a realistic comparison of results within or between centers. Johanson et al. [8] reviewed six acetabular defect classification systems and concluded that only one demonstrated the required reliability and validity for a standardised grading system. Most current classifications only roughly guide surgical plans which need to be decided based on specific intraoperative situations [3, 8, 9, 12]. Campbell et al. [7] claimed that the Paprosky system should be “considered only as a general guide” owing to its poor reliability. Consequently, the degree of bone defects found during surgery may exceed the surgeon’s expectations. However, altering the surgical plan intraoperatively is difficult, and insufficient preparation may lead to surgical failure and negative postoperative outcomes. In addition, current classifications are useful in evaluating simple defects than complex cases [12]. Ghanem et al. [9] reported that 16.37% of cases had a greater extent of defects found intraoperatively than that in the preoperative plan. They suggested a practical, reproducible, and valid classification system for preoperative planning.

One reason for this is that the previous classifications are old, and therefore mostly based on outdated radiological techniques. It is difficult to fully comprehend 3D structures of bone defects using X-rays or CT models on 2D computer screens, thereby providing incomplete information [13–15]. With the advent of AM technology, the resulting models are able to display the 3D anatomical structure in greater detail and provide key information [16–20]. Thus, AM models aid surgeons in understanding bone defects in detail and formulating surgical plans more comprehensively, which reduce errors and improve postoperative outcomes compared with relying solely on X-rays or CT models [20].

For this reason, we proposed a 3D acetabular defect classification system aided with AM models. Since type I as per this classification was characterised by minor defects easily assessed using X-rays or CT, there was no need for printing AM models or performing reliability and validity tests for patients in this category.

This study showed that both classification results and proposed surgical plans had excellent inter- and intra-observer reliability and excellent validity compared with the surgical records. This suggests that our 3D classification provided accurate classification of acetabular defects preoperatively and consequently guided reliable surgical plans owing to the several advantages afforded by this system. First, the surgical team had access to comprehensive information, especially for complex cases, where the full extent of the bone defects and residual bone mass was visible in the AM models. Hence, the accurate representation of the defects reduced the demand for radiography readings. This was further reflected by high intra-observer reliability in the assessments by the three surgeons. Second, the anatomical structures displayed by AM models were highly detailed, such that the classifications by the surgeons were very similar across both assessments, explaining the high inter-observer reliability. Third, evaluations were based on an objective AM model, which could be used for surgery simulation to test the feasibility of the plan and reflect potential intraoperative challenges preoperatively. Thus, the classification results and surgical plans were highly valid. This is consistent with the results of previous reports stating that preoperative planning with AM models before hip revision improved clinical results [16–20]. AM also improves diagnostic accuracy in the discovery of fractures in complex anatomical areas, such as obscure pelvic fractures [31, 32].

In this classification, the most important factor in consideration is achieving the initial stability when using a cementless cup, including rotational and vertical stability. The initial stability can be obtained either by effective three-point fixation spanning over 180°, or by screw fitting into the wall. We selected the anterosuperior acetabulum, ischial ramus, and pubic ramus for three-point fixation because other structures around the acetabulum are relatively thin and are, therefore, non-viable options in cases with severe defects. Despite residual bone mass, this type of bone has high density due to osteosclerosis, and is often brittle, and is, therefore, not a good option for fixation. The three areas we selected have a larger interface and sufficient bone mass, providing scope for fixation regardless of osteolysis. Although the ischial ramus and anterosuperior acetabulum have a relatively large bone stock, and some surgeons believe that these two points are sufficient to hold the cup, this approach should be avoided. In some cases categorised as type III, the defects of the anterosuperior acetabulum or ischial ramus can still be reconstructed with a buttress plate or augments, and a three-point fixation can be obtained by combining the other two locations. If more than one place requires reconstruction, the above method might not provide sufficient holding.

**Fig. 10:**
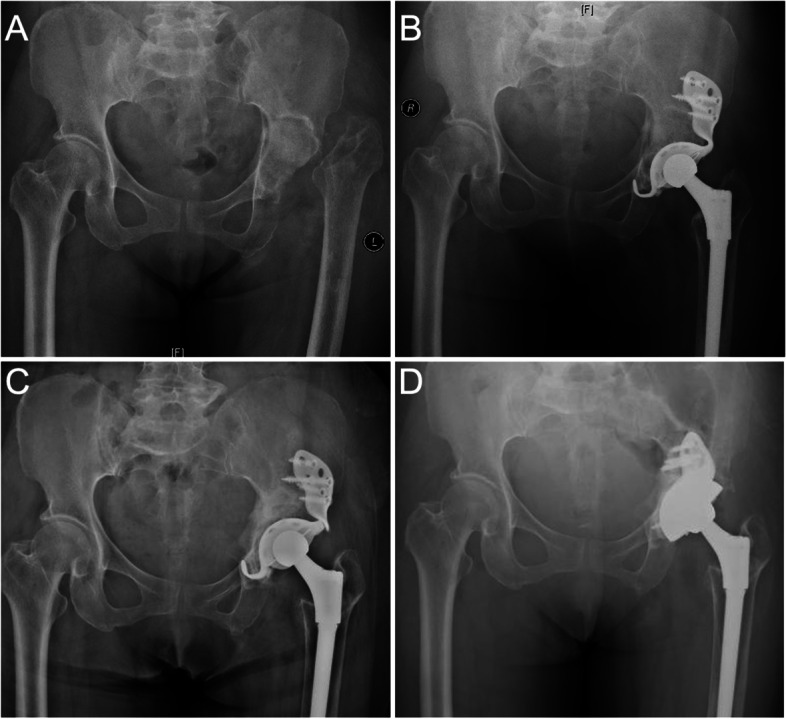
**A** Pelvis anteroposterior radiograph taken before the first operation. The left femoral neck was fractured. **B** Radiograph taken right after the primary THA and a commercial cage was used. **C** 7 years after the first operation, fatigue failure happened in the connection between the wing and cup. **D** A high friction coefficient revision cup was used in revision

We classified cases with internal acetabular wall defects but an intact acetabular ring (Paprosky type 2C) as type II because even if the remaining bone is sufficient to provide a three-point fixation, it is still recommended that the cup be held with augments or screws. If type II defects are present in the stress-bearing posterosuperior acetabulum, we believe that direct support between the bone and prosthesis is essential for achieving reliable long-term stability through bone remodeling. When structural or compression bone grafting is used, the process of bone remodeling is prolonged, resulting in weight-bearing at the interface between the prosthesis and graft. The prosthesis rapidly loosens as a result of micromotion. Our cases demonstrated that in the cases of no direct contact between the prosthesis and host bone and contact mediated solely through an allograft, fatigue fractures occurred in the connection part to the cage due to micromotion even though the allografts were well-constructed (Fig. 10). It is possible that the remodeling progress was so long that the grafted bone could not provide sufficient support to the prosthesis in the weight-bearing area, despite the grafted bone being well remodeled.

In type II defects with cavities or uncontained defects in the weight-bearing posterosuperior acetabulum, augments are needed. When the bone defects are not severe and host bone thickness is sufficient, conventional commercial augments can repair the defects, and the cup can be fixed by the remaining acetabulum wall or screws. Stable fixation cannot be achieved when uncontained or severe defects are present. Despite bone mass in the adjacent ilium in some cases, it is difficult to directly fix an augment onto it. Custom augmented fixed wings or buttress plates were required.

In clinical practice, we observed few patients with defects only in the anterosuperior acetabulum, potentially because little force was exerted on the anterosuperior region. Anterosuperior defects usually occur in conjunction with posterosuperior defects, resulting in defects in the superior acetabulum, categorised as type III. When severe osteolysis occurs only in either the superior acetabulum or ischial ramus, a buttress plate or customized augment with a wing can be used to reform their anatomies with the ability to hold the cup. When more than two of these locations are defective, the initial vertical and rotational stabilities cannot be achieved using conventional methods. Cup-cages or customized prostheses are recommended. Currently, most commercial cup-cages are used when there are no serious bone defects at the bottom acetabulum. The cup-cage can be fixed using an obturator hock into the obturator foramen or a hook bound to the upper edge of the obturator. Customized cages can be used for defects in areas where rotational stability is difficult to obtain.

However, it is important to note that this study has certain limitations. First, the original size of each patient’s pelvis and residual bones are different, and it is difficult to classify quantitatively. However, using this qualitative 3D classification, professional surgeons with expertise in joint revisions can already distinguish the extent of the bone defect and design feasible surgical plans. Second, the accuracy of measuring the effective bone mass by AM models requires further improvement [16]. However, the current accuracy meets the needs of most clinical applications. We recommended this surgical strategy, but acknowledge that experienced surgeons might have different approaches to these reconstruction methods.

## Conclusion

Our 3D acetabular defect classification aided with AM model has excellent reliability and validity. With this classification and the use of objective AM models, surgeons can evaluate bone defects intuitively and make more accurate surgical plans. This classification is a promising tool for surgeons for preoperative evaluation.

## Data Availability

The datasets used or analysed during the current study are available from the corresponding author upon reasonable request.

## Abbreviations

ICC: Intra-class correlation coefficient
2D: Two-dimensional
3D: Three-dimensional
AM: Additive manufacturing
CT: Computed tomography
AAOS: American Academy of Orthopaedic Surgeons
